# Can medical learners achieve point-of-care ultrasound competency using a high-fidelity ultrasound simulator?: a pilot study

**DOI:** 10.1186/2036-7902-5-9

**Published:** 2013-11-19

**Authors:** Adam R Parks, Paul Atkinson, Glenn Verheul, Denise LeBlanc-Duchin

**Affiliations:** 1 Dalhousie Medicine New Brunswick, 100 Tucker Park Road, Saint John, New Brunswick E2L 4 L5, Canada; 2 Emergency Department, Saint John Regional Hospital, 400 University Ave, Saint John, New Brunswick E2L 4 L4, Canada; 3 Memorial University, 230 Elizabeth Ave, Saint John, Newfoundland A1B 3X9, Canada; 4 Department of Psychology, University of New Brunswick Saint John, 100 Tucker Road, Saint John, New Brunswick E2L 4 L5, Canada

**Keywords:** Emergency medicine, Simulation, Medical education, Ultrasound, Echocardiography

## Abstract

**Background:**

Point-of-care ultrasound (PoCUS) is currently not a universal component of curricula for medical undergraduate and postgraduate training. We designed and assessed a simulation-based PoCUS training program for medical learners, incorporating image acquisition and image interpretation for simulated emergency medical pathologies. We wished to see if learners could achieve competency in simulated ultrasound following focused training in a PoCUS protocol.

**Methods:**

Twelve learners (clerks and residents) received standardized training consisting of online preparation materials, didactic teaching, and an interactive hands-on workshop using a high-fidelity ultrasound simulator (CAE Vimedix). We used the Abdominal and Cardiothoracic Evaluation by Sonography (ACES) protocol as the curriculum for PoCUS training. Participants were assessed during 72 simulated emergency cardiorespiratory scenarios. Their ability to complete an ACES scan independently was assessed. Data was analyzed using R software.

**Results:**

Participants independently generated 574 (99.7%) of the 576 expected ultrasound windows during the 72 simulated scenarios and correctly interpreted 67 (93%) of the 72 goal-directed PoCUS scans.

**Conclusions:**

Following a focused training process using medical simulation, medical learners demonstrated an ability to achieve a degree of competency to both acquire and correctly interpret cardiorespiratory PoCUS findings using a high-fidelity ultrasound simulator.

## Background

Over the past two decades, bedside point-of-care ultrasound (PoCUS) has evolved into an important adjunct to clinical examination in acute care specialties such as emergency medicine, intensive care medicine, and internal medicine. The versatility of PoCUS makes it an ideal imaging modality in the evaluation of the critically ill patient. Multiple etiologies can be considered and investigated, all with the same machine, in a matter of minutes. Incorporation of goal-directed PoCUS in early patient management improves diagnostic accuracy, shortens the list of viable diagnostic etiologies, and changes treatment plans for certain emergency pathologies [1-3].

Goal-directed PoCUS protocols have been developed to provide a structured approach to improving the diagnostic accuracy of the initial clinical assessment of the critically ill patient and to monitor fluid resuscitation. Two common examples of these protocols are the 'abdominal and cardiac evaluation with sonography in shock’ (ACES) protocol, proposed by one of the authors (Atkinson et al.) in 2009 [1], and the 'rapid ultrasound for shock and hypotension’ (RUSH) protocol [4]. These goal-directed scans aim to provide a structured approach to PoCUS in hypotension and require only an introductory level of training.

The ACES protocol describes a structured six-view scan involving a (1) cardiac view, (2) inferior vena cava (IVC) view, (3) abdominal aorta view, (4) right and (5) left flank views for intra-abdominal and pleural fluids, and (6) a pelvic view for free fluid as a helpful adjunct to clinical examination. The addition of appropriate windows for pneumothorax and DVT are advised where considered appropriate.

The limitation with PoCUS, as with many aspects of the physical exam and other bedside tests, is that ultrasound is operator dependent. Confidence and competence in both image acquisition and image interpretation is essential, as is the knowledge of how to incorporate findings into clinical decision-making. Given the increased usage and significance of PoCUS in clinical medicine [5] and the importance of ensuring that residents and students are familiar and competent with ultrasound, the purpose of this study is to evaluate the use of high-fidelity simulation to train medical learners in PoCUS.

Medical training using simulation is a well-established and respected practice [6,7] and has many advantages when compared to traditional training techniques. Traditional medical teaching can be reliant on patient and pathology availability, and safety issues. With simulation, learners can be exposed to almost any clinical scenario as many times as needed without any consequence towards patient health. In this respect, simulator-based learning can provide a much more efficient and safer way of training inexperienced medical students, as well as seasoned physicians.

The breadth of simulator research is noteworthy [8]. However, to the best of our knowledge, there is little research done in this field that focuses on the evaluation of PoCUS and using simulators in training medical students and resident physicians to diagnose emergency cardiac and pulmonary pathologies. As such, this study includes certain methodologies that are considered exploratory: the training and testing protocol itself, along with the employment of PoCUS with patient simulators.

Our primary objective is to assess whether the focused training process described in this study is effective in training medical students and resident physicians to competently perform a goal-directed PoCUS scan in a simulated setting. In this study, competency is defined as the participants' ability to acquire and interpret PoCUS images, as judged by an experienced emergency physician. We hypothesized that the ACES protocol could be learned on a high-fidelity simulator, and integrated into standardized emergency medical scenarios, by medical trainees with minimal or no prior ultrasound experience. As a secondary objective, information was collected that focused on participant confidence levels, as well as face and content validity of the PoCUS simulator.

## Methods

### Study design

This was a prospective observational study involving medical school clerks (third- and fourth-year medical students) and resident physicians (PGY1-3) completing rotations in an urban regional hospital. The study was approved by the Dalhousie University Research Ethics Board.

### Study setting and population

The study was conducted in the simulation center located in a multidisciplinary teaching facility at the New Brunswick Community College between March 2012 and September 2012. All 12 participants enrolled voluntarily in the study after recruitment using local communication channels and signed a consent form agreeing to keep all aspects of the study confidential. Inclusion criteria were (a) voluntary participation, (b) being available and willing to attend the training and testing session, and (c) being either a third- or fourth-year medical student clerk *or* a physician currently in their first 3 years of residency training. Participants were included in the study regardless of prior ultrasound training.

### Study protocol

The study protocol was broken up into three sections: (1) preparation, (2) training, and (3) testing. After each stage, participants were given a 5-point Likert scale questionnaire regarding their confidence in diagnosing emergency disease using PoCUS. At the end of the session, participants were also given a questionnaire regarding the face and content validity of the PoCUS simulator.

**Figure 1:**
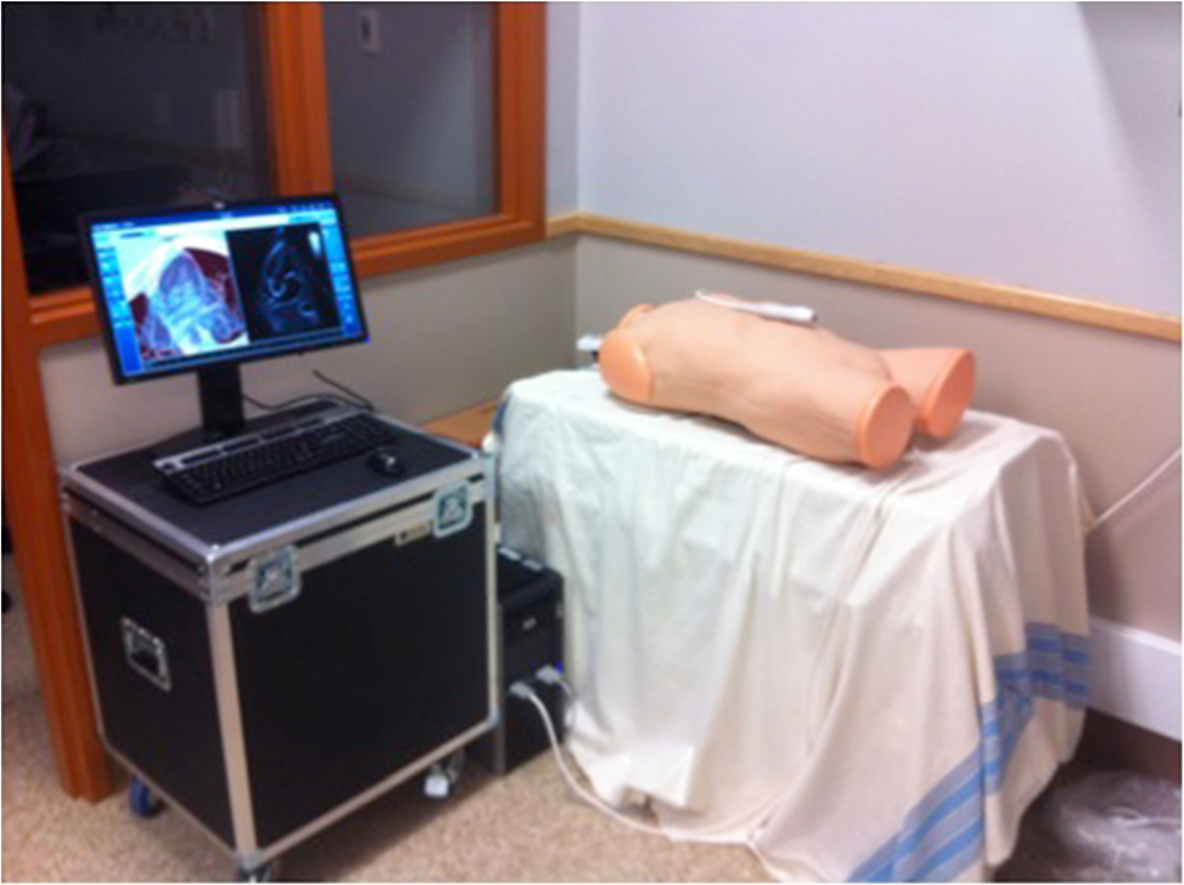
Picture of the CAE Vimedix ultrasound simulator.

1. Preparation consisted of seven brief video clips and two pre-readings. Preparation materials were given to participants at least 3 days prior to the participants' session date. The video clips included typical abdominal and cardiac ultrasound views both with and without pathology. The readings included the Emergency and Critical Care Ultrasound Course introductory manual [9], which introduced the fundamentals and physics behind ultrasound, as well as an overview of the core applications of PoCUS used in this setting, and also a published summary of the ACES scan [1] which was the goal-directed PoCUS scan chosen for this study. The ACES scan is recognized internationally and was chosen ahead of other goal-directed scans due to local availability of certified teachers through the Emergency Critical Care Ultrasound course.

2. The PoCUS training session occurred in groups of one to three participants and began with a standardized, 1-h, focused lecture. This lecture introduced the fundamentals of PoCUS and focused on the details surrounding the ACES scan and how to identify certain pathologies. This was followed by hands-on PoCUS training, which involved a 1-h session with the CAE Vimedix PoCUS simulator (Figure 1). On the PoCUS simulator, participants were able to observe an ACES scan performed by the instructor and were then able to practice the ACES scan individually. Various pathologies were also presented to participants on the PoCUS simulator, none of which were included in the testing procedure. Once participants felt comfortable scanning on the simulator, they then proceeded to testing.

3. Testing consisted of a set of six emergency medical scenarios for each participant, each with pathology that was potentially detectable by PoCUS. The scenarios were aortic stenosis, cardiac tamponade, dilated cardiomyopathy, myocardial infarction (MI), pleural effusion, and pulmonary embolism (PE) (features of the PoCUS simulator for each scenario are included in the Appendix). Each of the 12 participants completed all six scenarios, giving a total of 72 data sets. The scenarios were counterbalanced using a Latin square design to ensure that each participant underwent a different sequence of scenarios. This arrangement was intended to minimize carryover effects due to practice and learning. For every scenario, the simulation suite was equipped with the PoCUS simulator (programmed for the given scenario) and an emergency physician, who observed each PoCUS scan. Participants were instructed only to consider the patient's diagnoses and were not expected to provide any interventions or treatment options for the simulated patient. Participants were not aware of the diagnoses at any point prior to or during the scenarios.

During the PoCUS scan, the participant was observed by an emergency physician certified to teach PoCUS. The observing physician graded the participant on whether they acquired all relevant views. Using the information gathered with the PoCUS simulator, the participant interpreted and recorded their ultrasound findings, noting any abnormalities they observed.

This method of testing was repeated for each participant until all six scenarios had been completed. Following the final scenario, participants reviewed each case in detail with the instructor.

### Technical specifications

The CAE Vimedix (CAE Healthcare, Saint-Laurent, Quebec, Canada) PoCUS simulator was used as the bedside ultrasound simulator. All scenarios corresponded to CAE-programmed pathology cases and were used in stealth mode (diagnostic labels were removed).

### Measurements

An emergency physician, certified in teaching PoCUS, observed and evaluated each participant for proper image acquisition using a standardized checklist. PoCUS scans were defined as complete or incomplete, with specific omissions being recorded.

Following each PoCUS scan, participants would record their interpretation of the scan on a standardized sheet. The investigators then reviewed the interpretations to determine accuracy.

In addition, in order to aid in future studies, confidence questionnaires and face and content validity questionnaires were collected. The confidence questionnaires were collected following preparation, training, and testing. These questionnaires measured the participant's confidence in diagnosing emergency pathologies using bedside ultrasound. The questionnaires used a 5-point Likert scale (1 = extremely unconfident, 3 = neutral, 5 = extremely confident). Questionnaires regarding the simulators' face and content validity were collected from participants following testing. Face and content validity questionnaires used questions similar to those described by Weidenbach et al. [10] to gauge the participants' views on the realism of the PoCUS simulator and how effective it was in teaching proper PoCUS diagnostic technique. These questionnaires also employed a 5-point Likert scale (1 = not at all, 3 = neutral, 5 = very much).

### Data analysis

All data analysis was completed using R software, version 2.15 (R Foundation for Statistical Computing, Vienna, Austria). Descriptive statistics were used to summarize response to the confidence and the face and content questionnaires.

## Results

A total of 12 medical learners were recruited to participate in the study (six third- and fourth-year Dalhousie University medical student clerks and six PGY1-3 resident physicians training in the Saint John area).

No participant had previously received any formal ultrasound or echocardiography training. All 12 learners completed the necessary preparation before attending their session, and all learners completed training and testing during the day of their session. Each participant completed all six scenarios. Participants had a minimum of 3 years of medical training, including classroom and clinical emergency medicine rotations focused around diagnosing emergency cardiorespiratory pathologies. There were no indeterminate results, missing response, or adverse events recorded throughout the entirety of the study.

### Image acquisition

Although the published ACES scan consists of six views, in this study, the two right and left 'flank views’, which typically consist of abdominal fluid and pleural views, were considered four separate views in order for observers to be more specific with their feedback. As such, each ACES scan consisted of eight views (i.e., cardiac view, IVC view, abdominal aorta view, right and left flank views for intra-abdominal fluid, right and left lung base views for pleural fluid, and a pelvic view for free fluid). With 72 scans being completed, a total of 576 ultrasound windows were obtainable. Of the possible 576 ultrasound windows, 574 (99.7%) were correctly acquired. One participant, during one scenario, did not complete a full ACES scan, in that they failed to obtain adequate views of both lung bases.

### Image interpretation

The majority of goal-directed PoCUS scans were interpreted correctly. Out of 72 scans, 67 (93%) had recorded accurate ultrasound findings. These five false-negative scans were all interpreted as 'normal’ or 'nil’ for all participants. The distribution of false-negative scans for each scenario is described in Table 1, along with sensitivities and 95% confidence intervals.

**Table 1:** Distribution of emergency scenarios with corresponding test interpretations and 95% CIs

**Diagnosis**	**Interpretation**		**Test sensitivity (%) (95% CI)**
	**+**	**-**	
Aortic stenosis	12	0	100 (73.4 to 100)
Tamponade	12	0	100 (73.4 to 100)
Myocardial infarction	11	1	91.67 (61.5 to 98.6)
Pleural effusion	10	2	83.33 (51.6 to 97.4)
Pulmonary embolism	11	1	91.67 (61.5 to 98.6)
Dilated cardiomyopathy	11	1	91.67 (61.5 to 98.6)

### Questionnaires

The questionnaires were easily administered and provided relevant, helpful information. As only 12 sets of questionnaires were collected (one set of questionnaires per participant), it was not possible to make any statistically significant conclusions regarding changes in participant confidence or simulator face and content validity.

Participants' median confidence levels in diagnosing emergency pathologies using PoCUS were 'very unconfident’ (1.0 out of 5) before training, 'neutral’ (3.0 out of 5) after training, and 'somewhat confident’ (4.0 out of 5) after testing. Results from the face validity questionnaire indicated that the general realism of the simulator was viewed as 'somewhat realistic’ (median response, 4.0 out of 5), the realism of the ultrasound image was viewed as 'somewhat realistic’ (median response, 4.0 out of 5), and the realism of the presented pathologies was viewed as 'somewhat realistic’ (median response, 4.0 out of 5). In addition to this, participants' median responses indicated that the simulator could effectively teach PoCUS techniques (median response between 'somewhat’ and 'very much’, 4.5 out of 5) and could make a significant contribution to quality assurance (median response 'very much’, 5.0 out of 5).

## Discussion

This study demonstrated that medical learners with no previous formal training in ultrasound achieved a degree of competency in simulated PoCUS, following a focused training approach incorporating high-fidelity simulation using a goal-directed PoCUS scan (ACES). The brief training protocol presented in this study provides learners with the ability to effectively acquire and interpret ultrasound images.

With traditional training techniques, image knowledge and interpretation can rely heavily on didactic teaching. Our training protocol employed self-directed and didactic teaching techniques along with a PoCUS simulator, providing medical learners the opportunity to not only interpret ultrasound images but also acquire them. The use of a PoCUS simulator in training has previously been shown to be more effective in teaching medical learners to correctly interpret 'focused assessment with sonography in trauma’ (FAST) scan windows when compared to classroom-based learning [11].

Only one participant during one scenario was unable to acquire all the necessary ultrasound windows. The windows that were missed were the right and left lung bases. This occurred during the pleural effusion scenario. Since pleural effusions present findings at the lung bases, the participant was unable to accurately interpret and integrate the ultrasound knowledge. This example perhaps demonstrates the importance of ensuring that learners have a solid foundation in how to acquire ultrasound images before focusing on interpretation and clinical integration.

Image interpretation data indicated that participants were competent at identifying the majority of pathologies, especially those pathologies with apparent sonographic findings. Aortic stenosis and tamponade scenarios all returned with accurate interpretations by participants, whereas pathologies with more subtle sonographic findings, such as MI and PE, had one to two false negatives.

Although no statistical comparisons could be made, the questionnaires were easily administered and collected useful information regarding the simulators and participants' confidence in diagnosing emergency pathologies and incorporating PoCUS into their diagnosis.

The current study demonstrated that medical learners (medical students and residents) can quickly assimilate the required knowledge and hands-on skills to perform a focused PoCUS protocol (ACES) using a high-fidelity ultrasound simulator. Recommended training for PoCUS involves initial induction (introducing image generation and interpretation, and how to apply this to patient care), gaining experience (in emergency applications), and competency (gaining the ability to proficiently perform a PoCUS scan and adjust patient care accordingly). After minimal preparation, a short lecture, and experience with the PoCUS simulator, participants received a focused introduction to PoCUS scans and gained significant experience in PoCUS simulation. The focused and practical nature of this training protocol would be useful during clerkship and residency training programs.

### Limitations

Baseline data regarding participants and their experience with PoCUS and simulation was not formally assessed. Although direct questioning confirmed that no participant had any formal ultrasound training, this baseline data could have had a significant effect on the measured outcomes in this study and in future studies.

Participants were volunteers from a large pool of medical learners. This can create a self-selection bias in which only highly motivated learners participated. In addition, the high-fidelity PoCUS simulator used in this study is a relatively new simulator. As such, there is little data available that demonstrates its face, content, and construct validity as a simulator.

Long-term retention of knowledge and competency were not tested in this study. In addition, transfer of this competency into a clinical setting was not evaluated.

### Future research and follow-up

We plan to assess and report the diagnostic impact of the use of a simulated PoCUS protocol (ACES) during standardized simulated medical emergencies with the current study’s participants. We also hope to follow our participants to determine the extent of their long-term retention of this training and knowledge, especially in terms of their ongoing training and clinical implementation of PoCUS. Future research is required to determine if the effects demonstrated in this study can be translated into a real-life clinical setting from the simulation setting.

## Conclusions

We have shown that after a brief training process, medical learners achieved a degree of competency to acquire and interpret PoCUS images using a high-fidelity ultrasound simulator (CAE Vimedix). Following image acquisition, participants demonstrated the ability to interpret their findings.

## Appendix

Features of the PoCUS simulator for each scenario

Scenario #1: 'Aortic Stenosis’

•Instructions:

○ Load 'Aortic Stenosis’ *stealth mode* on ultrasound, remove bowel gas.

Scenario #2: 'Cardiac Tamponade’

•Instructions:

○ Load 'Tamponade’ *stealth mode* on ultrasound, remove bowel gas.

Scenario #3: 'Myocardial Infarction’

•Instructions:

○ Load 'Acute Lateral Myocardial Infection’ *stealth mode* on ultrasound, remove bowel gas.

Scenario #4: 'Right Pleural Effusion’

•Instructions:

○ Load 'Right Pleural Effusion’ *stealth mode* on ultrasound, remove bowel gas.

Scenario #5: 'Pulmonary Embolism’

•Instructions:

○ Load 'Pulmonary Hypertension’ *stealth mode* on ultrasound, remove bowel gas.

Scenario #6: 'Dilated Cardiomyopathy’

•Instructions:

○ Load 'Dilated Cardiomyopathy with Severe Biventricular Dysfunction’ *stealth mode* on ultrasound, remove bowel gas.

## Abbreviations

ACES: Abdominal and cardiac evaluation with sonography in shock; FAST: focused assessment with sonography in trauma; MI: Myocardial infarction; PE: Pulmonary embolism; PoCUS: Point-of-care ultrasound; RUSH: Rapid ultrasound for shock and hypotension.

## Competing interests

The authors declare that they have no competing interests.

## Authors' contributions

AP and PA contributed to the project design, ethics application, data acquisition and analysis, and final project composition. GV contributed to the project design and data acquisition. DLD contributed to the project design and final project composition. All authors read and approved the final manuscript.

## Authors' information

AP is a medical student at Dalhousie Medicine New Brunswick. PA is an emergency physician and director of research in the emergency department at the Saint John Regional Hospital, an associate professor at Dalhousie University and Memorial University of Newfoundland, and co-director of Emergency and Critical Care Ultrasound Canada. GV is an emergency physician, associate professor, and director of professional development at Dalhousie University, Memorial University of Newfoundland, and the Saint John Regional Hospital. DLD is an applied health coordinator at Horizon Health Networks in New Brunswick.
